# Slowed Intestinal Transit Induced by Less Mucus in Intestinal Goblet Cell Piezo1-Deficient Mice through Impaired Epithelial Homeostasis

**DOI:** 10.3390/ijms241814377

**Published:** 2023-09-21

**Authors:** Feifei Fang, Ying Liu, Yilin Xiong, Xueyan Li, Gangping Li, Yudong Jiang, Xiaohua Hou, Jun Song

**Affiliations:** Department of Gastroenterology, Union Hospital, Tongji Medical College, Huazhong University of Science and Technology, Wuhan 430022, China; m202175789@hust.edu.cn (F.F.); m202075816@hust.edu.cn (Y.L.); m201975641@hust.edu.cn (Y.X.); m202275933@hust.edu.cn (X.L.); ligangping@hust.edu.cn (G.L.); yudong1218@hust.edu.cn (Y.J.); houxh@hust.edu.cn (X.H.)

**Keywords:** Piezo1, intestinal transit, goblet cell, colon stem cell, intestinal epithelial homeostasis

## Abstract

Mucus secreted by goblet cells (GCs) may play an important role in intestinal transit function. Our previous study found that Piezo1 protein is essential for GC function; however, the effect of GC Piezo1 on intestinal transit function is unclear. Our study aimed to investigate the effect of Piezo1 in GCs on intestinal transit and the potential mechanism. We compared intestinal mucus, fecal form, intestinal transit time, intestinal epithelial cell composition, and stem cell function in WT and GC-specific Piezo1-deficient (Piezo1^ΔGC^) mice. Our results revealed a correlation between mucus and intestinal transit: the less mucus there was, the slower the intestinal transit. Piezo1 deficiency in GCs led to decreased mucus synthesis and also disrupted the ecological niche of colon stem cells (CSCs). Through organoid culture, we found that the capacity of proliferation and differentiation in Piezo1^ΔGC^ mouse CSCs was significantly decreased, which also led to a reduced source of GCs. Further studies found that the reduced Wnt and Notch signals in colon crypts might be the potential mechanism. These results indicated the importance of GC Piezo1 in intestinal transit function, which acts by maintaining the homeostasis of intestinal epithelial cells and mucus.

## 1. Introduction

Slowed intestinal transit can be induced by a variety of factors, such as dietary factors (reduced fiber intake), functional or organic intestinal disorders (irritable bowel syndrome-constipation, functional constipation, and colorectal cancer), systemic diseases (hypothyroidism), and medications (opioids) [1,2,3]. Intestinal transit plays an important role in the formation and excretion of feces, which is regulated by the enteric nervous system (controlled by autonomic, peripheral, and central nerves), smooth muscle, hormones (mainly 5-HT), flora, and possibly mucus [4,5]. In addition to acting as a barrier, intestinal mucus may play an important role in propelling food and feces. Mucus acts as a lubricant to encapsulate feces and reduces resistance to fecal evacuation [6,7]. Abnormal goblet cell (GC) function and reduced mucus have been reported in constipation animal models [8]. However, whether and how GC function affects intestinal transit and possible mechanisms are not clear.

The Piezo1 protein (a large three-bladed propeller-like transmembrane protein) is a mechanically sensitive nonselective ion channel that directly senses changes in cell membrane tension and changes the molecular conformation of channels to the open conformation, which transduces mechanical stimuli into the elevation of cytosolic Ca^2+^ and consequently influences cell responses [9,10]. Piezo1 has been shown to regulate various biological processes such as vascular remodeling [11], bone formation [12], immune cell activation [13,14], axon regeneration [15], and aqueous humor outflow [16], which is associated with a variety of conditions such as osteoporosis [17], pancreatitis [18], and cancer (such as gastric cancer, lung cancer, bladder cancer, etc.) [19]. However, the role of Piezo1 in goblet cell and intestinal transit function remains unknown.

Intestinal epithelial homeostasis is the basis of normal intestinal function. As one of the major cell types in the intestinal epithelium, GCs play an important role in providing a protective barrier covering the intestine. The functions of GCs in mucus synthesis and secretion, antigen presentation, and flora regulation have been intensively studied in the past [20,21]. However, little attention has been paid to the impact of altered GC function on intestinal epithelial homeostasis.

The cKit-labeled GC subgroup at the base of colon crypts has been found to promote the maintenance of stem cell function, playing a role similar to Paneth cells in the small intestine [22]. The self-renewal character of intestinal stem cells (ISCs) results in abundant force alterations (stretch and extrude) in crypts [23]. As a mechanically sensitive ion channel, Piezo1 can regulate epithelial cell fate by recognizing stretch mechanical signals [24]. In our previous study, we demonstrated the importance of Piezo1 in GC mucin2 synthesis [2]. GCs (at the base of crypts) constitute the colon stem cell (CSC) niche. However, whether Piezo1 in GCs affects intestinal transit by regulating intestinal epithelial homeostasis remains unclear.

This study aimed to investigate the effect of GC Piezo1 on intestinal transit and the possible mechanisms affecting intestinal epithelial homeostasis. We compared intestinal mucus, fecal form, intestinal transit time, intestinal epithelial cell composition, and stem cell function in WT and GC-specific Piezo1 knockout (Piezo1^ΔGC^) mice. In addition, we preliminarily explored the possible molecular mechanisms of slowed intestinal transit in Piezo1^ΔGC^ mice.

## 2. Results

### 2.1. Decreased Goblet Cell Numbers and Thinner Mucus Layer in Piezo1^ΔGC^ Mouse Colons

**Figure 1:**
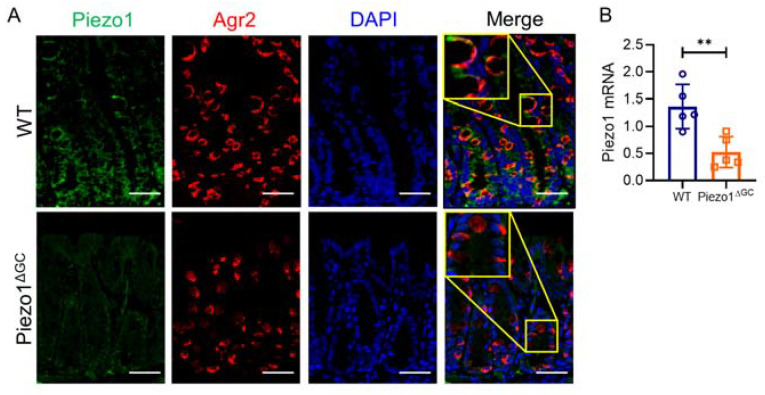
Identification of Piezo1^ΔGC^ mice. (**A**) Immunofluorescence co-localization of Piezo1 and Agr2 (label goblet cells) in WT and Piezo1^ΔGC^ mouse colons. The yellow box shows a magnified localized image. Scale bar: 100 μm. (**B**) mRNA level of *Piezo1* in WT and Piezo1^ΔGC^ mouse colon crypts (normalized to *GAPDH*). At least three independent experiments were conducted. Data are expressed as the mean ± SEM. (*n* = 5 mice). ** *p* < 0.01.

**Figure 2:**
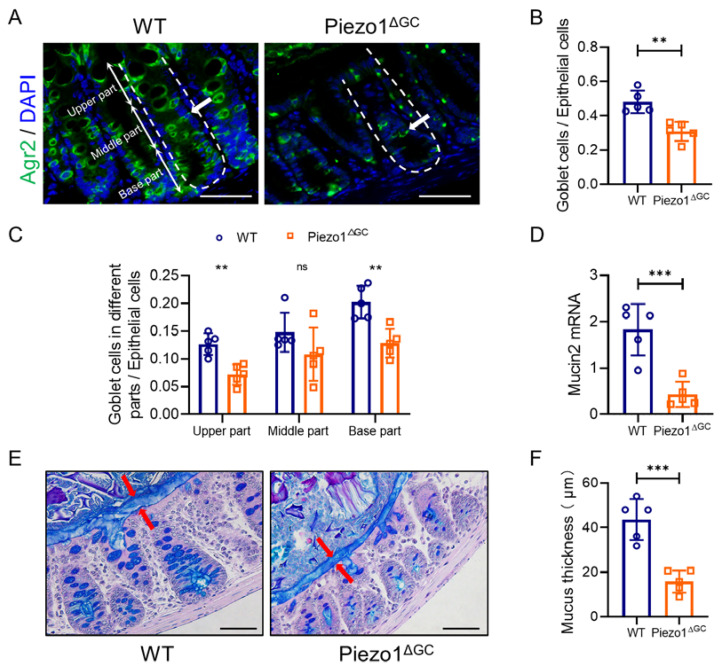
Decreased GC numbers and thinner mucus layer in Piezo1^ΔGC^ mouse colons. (**A**) Immunofluorescence staining of Agr2 in WT and Piezo1^ΔGC^ mouse colons. Scale bar: 100 μm. The crypt was divided equally into three parts: upper, middle, and base. The white arrow indicates a goblet cell. (**B**) Statistical analysis of goblet cells/epithelial cells in (**A**). (**C**) Statistical analysis of goblet cells in different parts/epithelial cells in (**A**). (**D**) RNA level of *Mucin2* in colon tissues from WT and Piezo1^ΔGC^ mice (normalized to *GAPDH*). (**E**) AB-PAS staining of mucus in WT and Piezo1^ΔGC^ mouse colons. The red arrows indicate the mucus layer. Scale bar: 50 μm. (**F**) Statistical analysis of mucus layer thickness in (**D**). At least three independent experiments were conducted. Data are expressed as the mean ± SEM. (*n* = 5 mice). ns, not significant; ** *p* < 0.01; *** *p* < 0.001.

We first generated Mucin2-CreERT2-Piezo1^flox/flox^ mice (Piezo1^ΔGC^ mice) and identified that Piezo1 on GCs was successfully knocked down by immunofluorescence (Figure 1A). The expression of *Piezo1* mRNA in Piezo1^ΔGC^ mouse colon crypts was decreased (Figure 1B). To determine the effect of Piezo1 deficiency in GCs, we quantified the changes in GC number and intestinal mucus. We found that the number of GCs (Figure 2A–C), the expression of *Mucin2* (Figure 2D), and the mucus thickness (Figure 2E,F) were significantly decreased in Piezo1^ΔGC^ mouse colons. The reduction of GCs was mainly in the top and bottom of the crypt (Figure 2C).

### 2.2. Decreased Fecal Excretion and Thinning Mucus Layer on the Fecal Pellet Surface in Piezo1^ΔGC^ Mice

**Figure 3:**
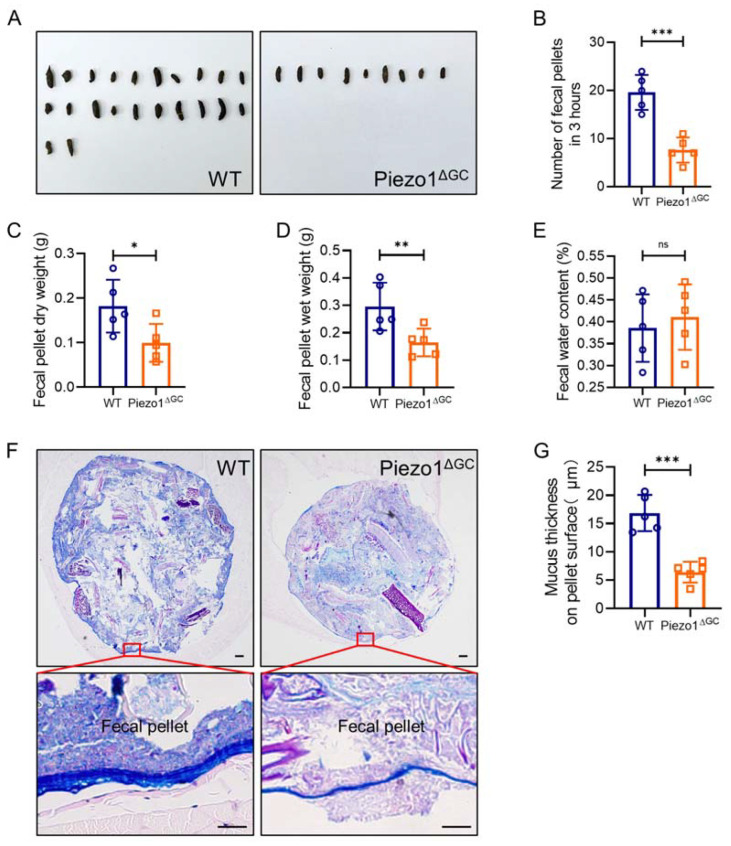
Decreased fecal excretion and thinning mucus layer on fecal pellet surface in Piezo1^ΔGC^ mice. (**A**,**B**) Fecal pellets excreted (**A**) and their number (**B**) in 3 h from WT and Piezo1^ΔGC^ mice. (**C**,**E**) Dry weight (**C**), wet weight (**D**), and water content (**E**) of feces excreted by WT and Piezo1^ΔGC^ mice in 3 h. (**F**) AB-PAS staining of mucus in and on the fecal pellet surface of WT and Piezo1^ΔGC^ mice. The fecal pellet from the distal colon was wrapped in the abdominal muscle. Scale bar: 100 μm. (**G**) Statistics analysis of mucus layer thickness in (**F**). At least three independent experiments were conducted. Data are expressed as the mean ± SEM. (*n* = 5 mice). ns, not significant; * *p* < 0.05; ** *p* < 0.01; *** *p* < 0.001.

Given that reduced intestinal mucus may influence fecal characteristics, we subsequently assessed defecation in mice. We found that fecal excretion in Piezo1^ΔGC^ mice per unit time (3 h) was significantly reduced (Figure 3A,B), and both the dry weight and wet weight of feces were also reduced (Figure 3C,D). In addition, although the feces excreted by the Piezo1^ΔGC^ mice were dry and hard, there was no significant difference in fecal water content between the two groups (Figure 3E). Furthermore, the mucus in and on the surface of feces isolated from the distal colon of the Piezo1^ΔGC^ mice was significantly reduced (Figure 3F,G), which may explain the dryness and hardness of the feces we observed in the Piezo1^ΔGC^ mice. Collectively, Piezo1^ΔGC^ mice excreted fewer feces than WT mice, and while the fecal pellets were smaller and encapsulated by a thinner coat of mucus, their water content was indistinguishable from the controls.

### 2.3. Prolonged Gastrointestinal Transit Time in Piezo1^ΔGC^ Mice Correlates with Intestinal Mucus Thickness

**Figure 4:**
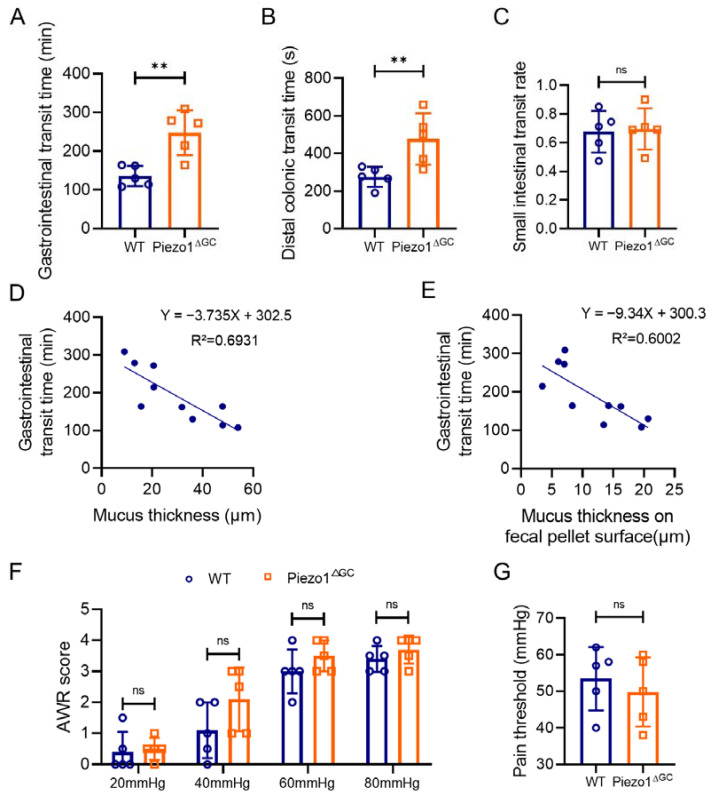
Prolonged gastrointestinal transit time in the Piezo1^ΔGC^ mice correlates with intestinal mucus thickness. (**A**–**C**) Intestinal transit-related parameters of WT and Piezo1^ΔGC^ mice: the gastrointestinal transit time (**A**), the distal colonic transit time (**B**), and the small intestinal transit rate (**C**). (**D**) Line regression of mucus thickness and gastrointestinal transit time. (**E**) Line regression of mucus thickness on fecal pellet surface and gastrointestinal transit time. (**F**,**G**) The AWR score and pain threshold measured by colorectal distension (CRD) test in the WT and Piezo1^ΔGC^ mice. At least three independent experiments were conducted. Data are expressed as the mean ± SEM. (*n* = 5 mice). ns, not significant; ** *p* < 0.01.

Mucus is an important intestinal content lubricant [5], and decreased intestinal mucus may lead to increased resistance to defecation. Thus, we subsequently evaluated the intestinal transit time in the WT and Piezo1^ΔGC^ mice. The gastrointestinal transit time and distal colonic transit time were significantly longer in the Piezo1^ΔGC^ mice (Figure 4A,B). The small intestinal transit rate was not obviously different between the two groups (Figure 4C). Notably, linear regression analysis suggested a negative correlation between mucus thickness and gastrointestinal transit time (Figure 4D,E). In addition, the Piezo1^ΔGC^ mice exhibited a constipated phenotype, but the visceral sensitivity had no obvious changes. The abdominal withdrawal reflex (AWR) score (Figure 4F) and pain threshold (Figure 4G) did not show any difference between the WT and Piezo1^ΔGC^ mice. Additionally, colonic smooth muscle contraction tests showed that the contraction amplitude, frequency, and motility index (MI) in isolated strips of colonic muscle from the Piezo1^ΔGC^ mice were not different compared to the controls (Figure S1A–C). These results suggested that the prolonged intestinal transit time in Piezo1^ΔGC^ mice was due to reduced intestinal mucus.

### 2.4. Abnormal Colonic Development in Piezo1^ΔGC^ Mice

**Figure 5:**
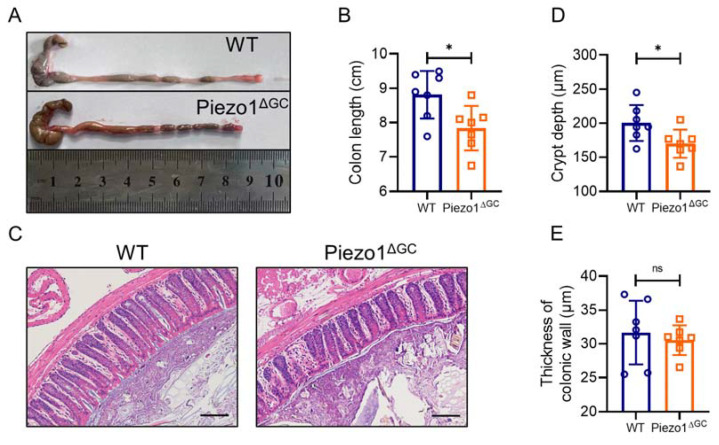
Abnormal colonic development in the Piezo1^ΔGC^ mice. (**A**) Gross image of colons from the WT and Piezo1^ΔGC^ mice. (**B**) Lengths of colons in the WT and Piezo1^ΔGC^ mice. (**C**) H&E staining of colon tissues in WT and Piezo1^ΔGC^ mice. Scale bar: 100 μm. (**D**) Depth of colon crypt in the WT and Piezo1^ΔGC^ mice. (**E**) The thickness of the colonic wall in the WT and Piezo1^ΔGC^ mice. At least three independent experiments were conducted. Data are expressed as the mean ± SEM. (*n* = 7 mice). * *p* < 0.05.

Next, we evaluated the effect on the overall development of the colon after Piezo1 deficiency in GCs. We found shorter colons (Figure 5A,B) and shallower crypt depths (Figure 5C,D) in the Piezo1^ΔGC^ mice, indicating abnormal colonic development. The thickness of the colon wall did not show a difference between the WT and Piezo1^ΔGC^ mice (Figure 5E).

### 2.5. Abnormal Intestinal Epithelial Cell Composition and Impaired Colon Stem Cell Niche in Piezo1^ΔGC^ Mice

**Figure 6:**
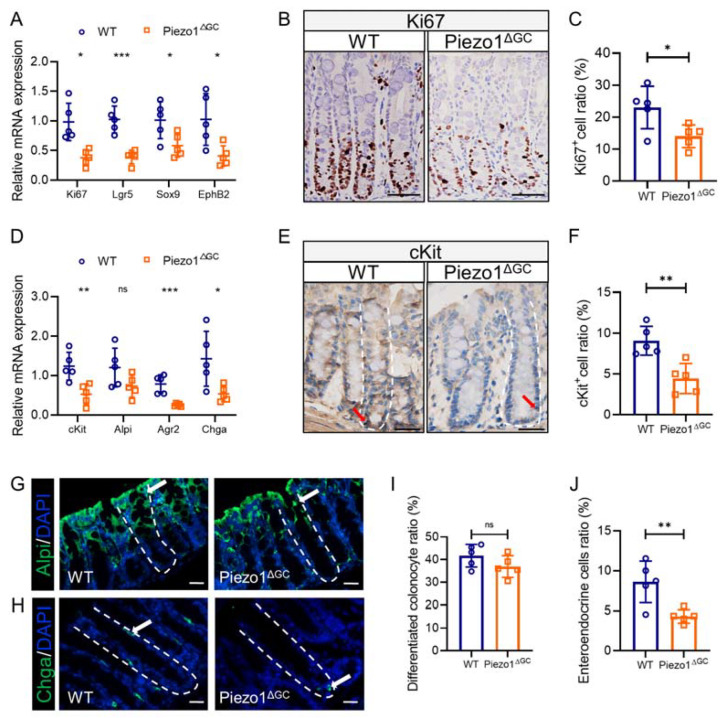
Abnormal intestinal epithelial cell composition and impaired colon stem cell niche in Piezo1^ΔGC^ mice. (**A**) RNA levels of *Ki67* and stem cell markers (*Lgr5*, *Sox9*, and *EphB2*) in WT and Piezo1^ΔGC^ mouse colon crypts (normalized to *GAPDH*). (**B**) Immunohistochemistry of Ki67 in WT and Piezo1^ΔGC^ mouse colons. Scale bar: 100 μm. (**C**) Statistical analysis of Ki67 positive cell ratio in (**B**). (**D**) RNA levels of stem cell niche marker (*cKit*), differentiated colonocyte marker (*Alpi*), goblet cell marker (*Agr2*), and enteroendocrine cell marker (*Chga*) in WT and Piezo1^ΔGC^ mouse colon crypts (normalized to *GAPDH*). (**E**) Immunohistochemistry of cKit in WT and Piezo1^ΔGC^ mouse colons. The white dashed lines mark the crypt borders, and the red arrow indicates a cKit-positive cell. Scale bar: 50 μm. (**F**) Statistical analysis of cKit-positive cell ratio in (**E**). (**G**,**H**) Immunofluorescence staining of Alpi (**G**) and Chga (**H**) in WT and Piezo1^ΔGC^ mouse colons. The white dashed lines mark crypt borders, and the white arrow indicates a differentiated colonocyte in (**G**) and an enteroendocrine cell in (**H**). Scale bar: 25 μm. (**I**,**J**) Statistical analysis of differentiated colonocyte ratio in (**G**) and enteroendocrine cell ratio in (**H**). At least three independent experiments were conducted. Data are presented as the mean ± SEM. (*n* = 5 mice). ns, not significant; * *p* < 0.05; ** *p* < 0.01; *** *p* < 0.001.

To explore the mechanism of reduced GCs and mucus, we subsequently examined the effect of Piezo1 deficiency in GCs on epithelial cell fate. We found that the colon crypts from the Piezo1^ΔGC^ mice contained fewer proliferating cells marked by Ki67 (Figure 6A–C). Meanwhile, the expression of colon stem cell markers (Lgr5, Sox9, and EphB2) was significantly reduced (Figure 6A). The cKit-positive GC subgroup at the base of colon crypts forms a stem cell niche that supports the growth of colon stem cells (CSCs) [22]. We found fewer cKit-positive cells in the Piezo1^ΔGC^ mouse crypts (Figure 6D–F), which means that there were a reduced amount of support cells around the CSCs. To assess intestinal epithelial cell differentiation, we quantified the number of differentiated surface colonocytes (labeled by Alpi) and enteroendocrine cells (EECs, labeled by Chga) in the colon crypts. The expression of *Chga* mRNA was significantly reduced in the Piezo1^ΔGC^ mice (Figure 6D). Consistent with the mRNA expression changes, we found fewer EECs in the Piezo1^ΔGC^ mouse crypts (Figure 6H,J). The proportion of differentiated surface colonocytes did not show a significant difference between the two groups (Figure 6D,G,I). Collectively, the disorder of the intestinal epithelial cell type in the Piezo1^ΔGC^ mice suggests impaired intestinal epithelial homeostasis.

### 2.6. Decreased Self-Renewal Capacity of Colon Stem Cells from Piezo1^ΔGC^ Mice

**Figure 7:**
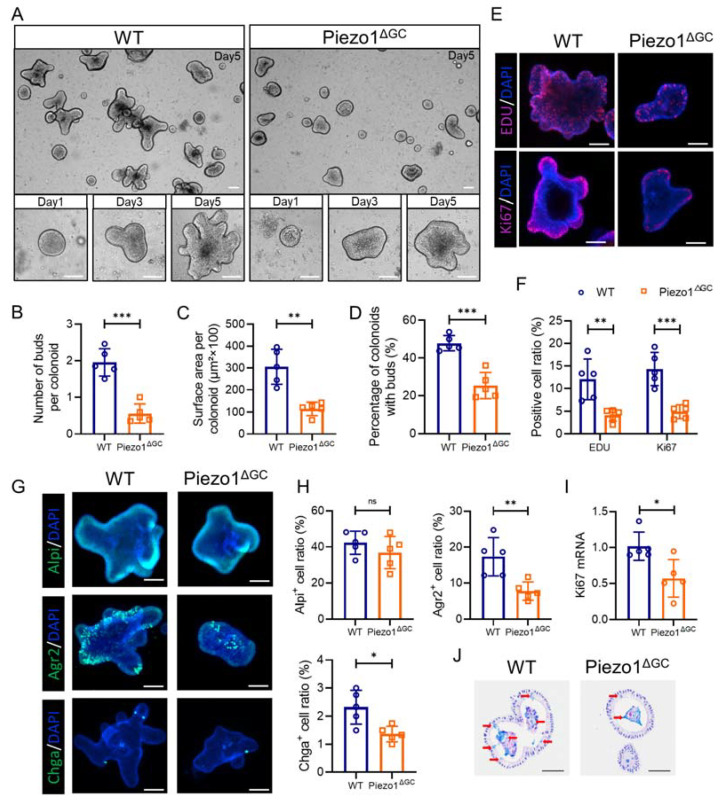
Decreased self-renewal capacity of colon stem cells from Piezo1^ΔGC^ mice. (**A**) Representative images of colonoids from WT and Piezo1^ΔGC^ mice at day 5. Images of one colonoid on day 1, 3, and 5 are below, reflecting the growth process of a colonoid. Scale bar: 100 μm. (**B**–**D**) Indicators related to colonoids growth: number of buds per colonoid (**B**), surface area per colonoid (**C**), and percentage of colonoids with buds per well (**D**). (**E**) Immunofluorescence staining of EDU and Ki67 in colonoids from WT and Piezo1^ΔGC^ mice. Scale bar: 100 μm. (**F**) Statistical analysis of EDU and Ki67 positive cell ratio in (**E**). (**G**) Immunofluorescence staining of differentiated colonocyte (Alpi), goblet cell (Agr2), and enteroendocrine cell (Chga) in colonoids from WT and Piezo1^ΔGC^ mice. Scale bar: 100 μm. (**H**) Statistical analysis of Alpi, Agr2, and Chga positive cell ratio in (**G**). (**I**) RNA levels of *Ki67* from WT and Piezo1^ΔGC^ mouse colonoids (normalized to *GAPDH*). (**J**) AB-PAS staining of colonoids from WT and Piezo1^ΔGC^ mice. The red arrows indicate mucus secreted by goblet cells in colonoids. Scale bar: 100 μm. At least three independent experiments were conducted. Data are presented as the mean ± SEM. (*n* = 5 mice). ns, not significant; * *p* < 0.05; ** *p* < 0.01; *** *p* < 0.001.

To further investigate the effect on CSCs after Piezo1 deficiency in GCs, we generated colonoids from the colon crypts of the WT and Piezo1^ΔGC^ mice. The size and budding of the colonoids reflect the self-renewal ability of the stem cells. At 5 days after seeding, we found that the colonoids from the WT mice had a larger surface area and more buds than the colonoids from the Piezo1^ΔGC^ mice (Figure 7A–C). The percentage of colonoids with buds was significantly reduced in the Piezo1^ΔGC^ mice (Figure 7D). In addition, the proliferating cells (indicated by EDU and Ki67) were significantly reduced in the colonoids from the Piezo1^ΔGC^ mice (Figure 7E,F). RT–PCR analysis showed a reduced expression of *Ki67* mRNA in the colonoids from the Piezo1^ΔGC^ mice (Figure 7I). Next, we examined the differentiation ability of the CSCs in vitro. Consistent with the in vivo results, the proportion of GCs and EECs in the colonoids from the Piezo1^ΔGC^ mice was significantly reduced (Figure 7G,H). The number of mucus-producing goblet cells was also significantly reduced in the Piezo1^ΔGC^ mouse colonoids (Figure 7J). Alpi-labeled differentiated colonocytes did not show obvious differences between the two groups. These data demonstrate that the abnormal epithelial stem cell niche caused by Piezo1 deficiency in GCs affects the self-renewal of CSCs, which might also lead to reduced GCs, slow crypt renewal, and delayed intestinal development.

### 2.7. Reshaped Wnt and Notch Signaling Expression in Piezo1^ΔGC^ Mouse Colon Crypts Leads to Reduced GCs

**Figure 8:**
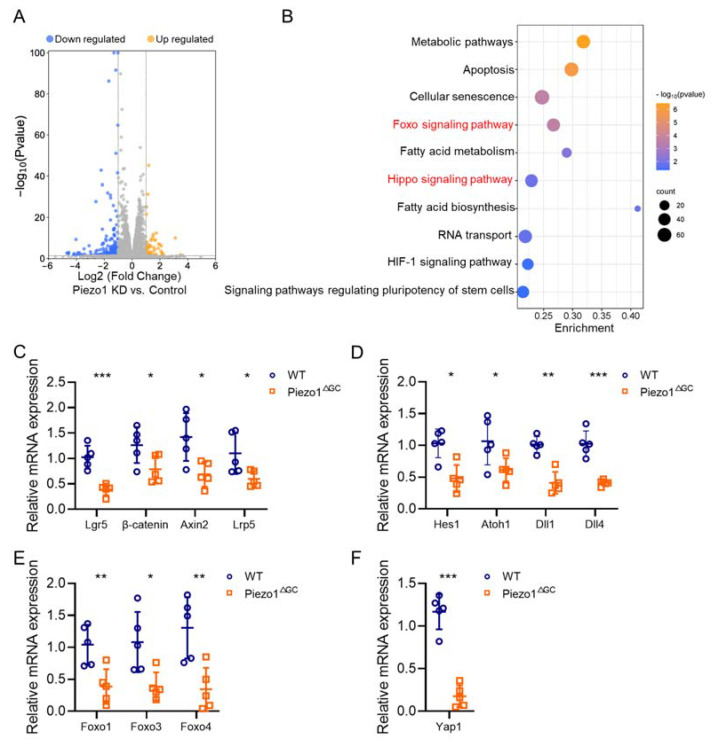
Reshaped Wnt and Notch signaling expression in Piezo1^ΔGC^ mouse colon crypts lead to reduced GCs. (**A**) Volcano plot depicting the differentially expressed genes between Piezo1-knockdown and Control LS174T cells (determined via RNA-Seq). The genes were selected via |Log2 (Fold Change)| > 1 and *p*-value < 0.05. The gray dots indicate genes with no significant difference between the two groups. (**B**) KEGG analysis of differentially expressed genes. The interested KEGG terms are marked in red. The threshold for significance was *p*-value < 0.05. (**C**,**D**) RNA levels of Wnt and Notch signaling pathway target genes in WT and Piezo1^ΔGC^ mouse colon crypts (normalized to *GAPDH*). (**E**,**F**) RNA levels of Foxo and Hippo signaling pathway key genes in WT and Piezo1^ΔGC^ mouse colon crypts (normalized to *GAPDH*). Data are presented as the mean ± SEM. (**A**, *n* = 3; **C**–**F**, *n* = 5 mice). ns, not significant; * *p* < 0.05; ** *p* < 0.01; *** *p* < 0.001.

A differential expression of Wnt and Notch signaling regulates intestinal stem cell (ISC) renewal and determines the fate of intestinal epithelial cells [25]. Given the significantly reduced renewal capacity of the CSCs following Piezo1 deficiency in GCs, we subsequently explored its effect on crypt Wnt and Notch signaling and its potential underlying mechanism. We constructed Piezo1-knockdown GCs (LS174T cell line) in vitro and performed RNA-Seq. LS174T cells were used to simulate the GCs [2]. A total of 274 differentially expressed genes were identified (Figure 8A). We did not find changed Wnt or Notch pathway key genes in the Piezo1-knockdown goblet cell line. However, we found significantly downregulated Wnt and Notch signals in the Piezo1^ΔGC^ mouse colon crypts (Figure 8C,D). Kyoto Encyclopedia of Genes and Genomes (KEGG) analysis revealed the enrichment of the Foxo and Hippo signaling pathways in the Piezo1-knockdown goblet cell line (Figure 8B), which reportedly crosstalk with Wnt and Notch signals in crypts to coregulate ISC growth [26]. Therefore, we also estimated the key genes of these pathways. RT–PCR analysis showed a reduced expression of key genes from the Foxo and Hippo signaling pathways in the Piezo1^ΔGC^ mouse colon crypts (Figure 8E,F). These results indicated that the remodeling of Wnt and Notch signals in the Piezo1^ΔGC^ mouse colon crypts may be attributable to changes in the Foxo and Hippo signals in GCs, which further affect the proliferation of CSCs and their differentiation into GCs.

## 3. Discussion

In summary, we have demonstrated the importance of GC Piezo1 in intestinal transit function and shown how it acts by maintaining the homeostasis of intestinal epithelial cells and mucus. Our results revealed a correlation between mucus and intestinal transit: the less mucus there was on the intestinal mucosal and fecal surface, the slower the intestinal transit. Piezo1 deficiency in GCs not only led to decreased mucus but also disrupted the ecological niche of CSCs. Through organoid culture, we found that the capacity of proliferation and differentiation in Piezo1^ΔGC^ mouse CSCs was significantly decreased, which also led to a reduced source of GCs and subsequent reduction of mucus. Furthermore, the disorders induced by Piezo1 deletion in the GCs resulted in abnormal intestinal development. Mechanistically, the reduced Wnt and Notch signals in the Piezo1^ΔGC^ mouse colon crypts may explain the decreased renewal capacity of the CSCs.

Mechanical signaling in the epithelial cells regulates cell fate [24]. We usually treat GCs as a separate group in crypts and investigate the role of Piezo1 in mucin synthesis. GC Piezo1 plays an important role in mucus synthesis and secretion, and our previous studies initially revealed how Piezo1 regulates GC function by downregulating histone methylation and promoting differentiation and maturation [2,27]. The expression of Atoh1, a factor that promotes GC maturation, was reduced in the colon crypts (Figure 8D). In addition, RNA-Seq showed the enrichment of apoptotic pathway genes in the Piezo1-knockdown goblet cell line (Figure 8B). Increased apoptotic signaling inhibits mucin synthesis in GCs [28]. The number of GCs was counted based on the immunofluorescence stains, and although not quantitative, it does show a decrease in the number of GCs. These results suggested that the decreased intestinal mucus in the Piezo1^ΔGC^ mice may have been a result of impaired maturation or increased apoptosis in the GCs.

On the other hand, the altered cell composition in the Piezo1^ΔGC^ mouse colon crypts suggested imbalanced homeostasis in the epithelium. Intestinal epithelial homeostasis requires the tightly balanced regulation of ISC self-renewal and differentiation [29]. The ISC itself, neighboring cells, and specific mesenchymal cells coregulate the ISC niche [30]. The impaired epithelial homeostasis may be triggered by the disruption of the stem cell niche in Piezo1^ΔGC^ mice (exhibiting decreased cKit^+^ GCs), which also led to reduced CSC renewal capacity. A significant reduction in GCs was found at the base of the crypts in the Piezo1^ΔGC^ mice (Figure 2C), which may explain the reduction in the CSC niche marker cKit. The weakened CSCs led to a reduced source of GCs and subsequently reduced mucus. In addition, it has been reported that epithelial cells can sense intercellular crowding to migrate upward and eventually undergo apoptosis in normal intestinal renewal. After inhibiting Piezo1, crowding-activated cell extrusion was blocked and affected epithelial cell renewal [31]. Considering that GCs in crypts are in an environment with rich mechanical signaling changes, we speculate that decreased GC sensitivity to mechanical forces in Piezo1^ΔGC^ mice led to blocked epithelial renewal and homeostatic imbalance. ISC function affects intestinal development, as has been observed in colitis mice with impaired ISCs and a shorter colon [32]. Thus, the decreased CSC renewal may be responsible for the shorter colons in the Piezo1^ΔGC^ mice.

More importantly, the results of our study indicate that the slowing of intestinal transit in the Piezo1^ΔGC^ mice was mainly associated with reduced intestinal mucosal and fecal surface mucus. Decreased mucus in the Piezo1^ΔGC^ mouse colon was due to the diminished function and reduced source of GCs. Our previous study showed that Yoda1 (Piezo1 agonist) intervention improved intestinal motility in WAS mice accompanied by increased mucus [2]. However, the action of Yoda1 may be a systemic effect, targeting not only the intestinal epithelium but also the Piezo1-positive enteric nervous system (ENS). Piezo1^ΔGC^ mice exhibited a slow transit constipation phenotype (prolonged intestinal transit time, reduced defecation, and reduced fecal water content) similar to those induced by loperamide [33]. The difference is that there was no decrease in the small intestinal transit rate or fecal water content in the Piezo1^ΔGC^ mice. The proportion of GCs was lower in the small intestine than in the colon (4%, 6%, 12%, and 16% of GCs in the duodenum, jejunum, ileum, and distal colon, respectively) [34]. Stool consistency in the small intestine was thinner [20]. Thus, the reduction in mucus in the Piezo1^ΔGC^ mice may not have been sufficient to cause a slowed small intestinal transit rate. On the other hand, the gastrointestinal transit time in the normal mice was about 120 min, and the distal colonic transit time in the normal mice was about 250 s. Both measures of transit time were significantly longer in the Piezo1^ΔGC^ mice (Figure 4A,B), seemingly indicating the role of goblet cell mucus in transit through the small intestine. There was no significant difference in the differentiated surface colonocytes, which were associated with colon water absorption between the two groups (Figure 6G). Therefore, we speculated that the drier and harder feces in the Piezo1^ΔGC^ mice were mainly associated with reduced mucus rather than decreased fecal water. Contrary to what we observed, another study showed more intestinal GCs in constipation patients than in normal controls [35]. However, this study did not quantify intestinal mucus, suggesting that the increased GCs may be compensative.

In addition, the AWR scores were not significantly different between the two groups, suggesting that the slowed intestinal transit in the Piezo1^ΔGC^ mice is not related to visceral sensitivity. Colonic smooth muscle contraction tests suggested that the slowed intestinal transit in the Piezo1^ΔGC^ mice was not caused by decreased muscle contractile ability. In our previous study, mucin-degrading bacteria such as Akkermansia muciniphila and Oscillospiraceae were decreased in Piezo1^ΔGC^ mouse colons, which indicated that a decrease in mucus was more likely caused by dysfunction in the GCs than mucus degradation due to flora disorder [21].

We explored the possible molecular mechanisms of reduced CSC renewal capacity in Piezo1^ΔGC^ mice. It is known that the Wnt and Notch signaling pathways control stem cell proliferation and differentiation during intestinal development. Typically, Wnt and Notch signaling act antagonistically in regulating intestinal cell differentiation fate, but the downregulation of Notch signals may result in the loss of Lgr5^+^ cells [36], which may lead to a simultaneous reduction in Wnt and Notch signals in intestinal crypts. Based on the absence of changes in the Wnt and Notch signals in the Piezo1-knockdown goblet cell line, we suggest that the reduced renewal ability of CSCs in Piezo1^ΔGC^ mice is associated with the downregulation of Wnt and Notch signals (caused by reduced Foxo and Hippo signals in crypts). The regulation of ISC stemness by the Foxo signaling pathway has been noted in the past [37]. Studies have reported crosstalk between Foxo and Wnt and Notch signaling in the intestine (i.e., the downregulation of Foxo1 and Foxo3 inhibits ISC proliferation and differentiation) [38]. In addition, Foxo1 can bind to the CSL element in the Hes1 promoter to promote neural stem cell differentiation [39]. Foxo3 regulates the expression of Notch1 and Notch3 receptors to promote skeletal muscle stem cell stemness [37]. The Hippo signaling pathway plays an important role in regulating development, tissue homeostasis, and regeneration [40]. The transcriptional coactivator Yes-associated protein 1 (Yap1) maintains ISC proliferation and stemness by interacting with β-catenin to activate Wnt signaling [41]. An agent-based simulation model has reported that the effect of Wnt signals can be influenced by the concentration of Hippo signals in crypts [25]. In hepatocytes, the activation of Yap1 upregulates Notch receptor expression, activating Notch signals and inducing the transposition of NICD to the nucleus [42]. These studies have reported crosstalk between the Hippo and Wnt/Notch pathways. However, how GC Piezo1 deficiency affects the delivery of Foxo and Hippo signals and the underlying mechanisms behind this are unclear, and we will explore this further in subsequent studies. 

Piezo1 mediates distinct Ca^2+^ signaling pathways to regulate cellular activities [9]. Yes-associated protein (Yap) has been shown to be an important downstream mechanotransduction effector of Piezo1 [43]. A recent study showed that the double-knockout of Yap/Taz in smooth muscle significantly downregulated the expression of colonic motility-related genes (such as Tpm1, Actg2, Acta2) and impaired colonic contractility, causing colonic pseudo-obstruction and lethality [44]. The reduced *Yap1* mRNA in the Piezo1^ΔGC^ mouse colon crypts (Figure 8F) suggested that intestinal GC Piezo1-knockdown may affect intestinal transit by inhibiting the Yap signaling pathway. However, we did not find significant changes in colonic smooth muscle in the Piezo1^ΔGC^ mice (including in the thickness of the colon wall and spontaneous activities), and the effect of reduced Yap1 in Piezo1^ΔGC^ mouse colons on intestinal motility needs to be confirmed in further studies. In addition, a previous study reported that intestinal epithelial Piezo1 knockout inhibited 5-HT synthesis and intestinal motility without disturbing the development of EECs [45]. This suggests that GC Piezo1 deficiency may also affect 5-HT levels in vivo, causing subsequent slowed intestinal transit; we will explore this in our future studies.

However, there are some limitations to our study. First, we failed to assess mucus in the small intestine due to its fluidity. In addition, due to the lack of methods targeting GCs to promote mucus secretion, we failed to demonstrate the effect of increased mucus on intestinal transit in vivo. Lastly, due to technical limitations, we did not detect the intercellular mechanical force changes in the Piezo1^ΔGC^ mouse crypts.

## 4. Materials and Methods

### 4.1. Mouse Model

Mucin2-CreERT2 mice (Mucin2-Tg mice) were crossed with Piezo1^flox/flox^ C57BL/6 mice (purchased from Shanghai Model Organisms Center, Inc., Shanghai, China) to generate the Mucin2-CreERT2-Piezo1^flox/flox^ mice (Mucin2-Tg-Piezo1^flox/flox^). The Mucin2-Tg-Piezo1^flox/flox^ mice were used as GC-specific Piezo1-knockout mice, and the Piezo1^flox/flox^ mice were used as the controls (WT). Then, tamoxifen was used to induce the deletion of GC Piezo1 in the Mucin2-Tg-Piezo1^flox/flox^ mice (Piezo1^ΔGC^ mice). For Piezo1 deficiency induction, 8-week-old mice were given 1 mg tamoxifen (dissolved in corn oil; intraperitoneal injection; Sigma-Aldrich, St. Louis, MO, USA) for 5 days. The controls were given the same volume of tamoxifen for 5 days. Observations and experiments were performed 7 days after the last induction. All mice were raised in the specific-pathogen-free (SPF) environment of the Animal Experimental Center of Tongji Medical College. The mice had free access to water and food. All animal experiments were approved by the Animal Experimentation Ethics Committee of Huazhong University of Science and Technology (IACUC Number: 2529).

### 4.2. Tissue Immunostaining

The distal colon tissues (2 cm from the anal) of each mouse were dissected and fixed with 4% paraformaldehyde (4% PFA). The fixed colon tissues were then embedded in paraffin and sectioned for subsequent experiments. The antibodies used in our experiments included the following: Piezo1 (1:200, 15939-1-AP, Proteintech, Chicago, IL, USA), Agr2 (1:200, AF6068, R & D Systems, Minnesota, USA), Ki67 (1:200, GB111141, Servicebio, Wuhan, China), cKit (1:150, ab256345, Abcam, Cambridge, England), Alpi (1:200, A0514, Abclonal, Woburn, MA, USA), and Chga (1:200, A9576, Abclonal).

For immunohistochemistry staining, the colon tissue sections were dewaxed with xylene, rehydrated with ethanols, and subjected to antigen retrieval via boiling in citrate buffer. Next, 3% H_2_O_2_ was treated for 5 min and then washed with PBS. A total of 30 min after blocking with 10% donkey serum, the primary antibody was incubated at 4° overnight. The secondary antibody (1:200, Antgene, Wuhan, China) was incubated the next day, and the nuclei were subjected to hematoxylin staining. The images were captured and viewed using a microscope (Olympus BX51, Olympus, Tokyo, Japan).

For immunofluorescence staining, after the same dewaxing and antigen retrieval procedures as mentioned above were carried out, the sections were permeabilized with 0.3% Triton for 15 min at room temperature, and subsequently blocked with 10% donkey serum for 1 h. Then, the sections were incubated in the primary antibody at 4° overnight. The next day, a secondary antibody was used for staining for 1 h at room temperature. The nuclei were then stained with 4′,6′-diamidino-2-phenylindole (DAPI, 1:1000; Antgene, ANT063) for 5 min. The images were captured and viewed using a fluorescence microscope (Axio Observer 7, Carl Zeiss, Oberkochen, Germany).

### 4.3. Alcian Blue Staining (AB-PAS Staining)

AB-PAS staining was performed in colonoids, colon tissues, and feces wrapped with abdominal muscle using methods that have been described previously [6]. The colonoids were made into sections before staining according to a method described previously [46]. The colonoids were stained to observe mucus-producing GCs. The colon tissue sections were stained to observe the entire mucus layer. The fecal sections were stained to observe the mucus layer on the surface of feces. Carnoy’s fluid-fixed colon tissue and feces were embedded in paraffin and made into sections. The Alcian Blue Periodic acid Schiff Kit (AB-PAS Kit) (Baso, Zhuhai, China) was used to carry out the staining, and the images were captured and viewed using a microscope (Olympus BX51, Olympus). We quantified the mucus layer thickness using ImageJ (V1.8.0, NIH, Bethesda, MD, USA) software in accordance with a previous study [6]. A total of 3–5 images per mouse were analyzed, and the values were averaged (5–10 measurements per image, n = 5 mice, 200×).

### 4.4. Hematoxylin and Eosin (H&E) Staining

After the same dewaxing and antigen retrieval procedures as mentioned above were carried out, the sections were stained with hematoxylin and eosin. The depths of the crypts and colonic walls were evaluated for each mouse (3–5 images) using ImageJ (V1.8.0, NIH, Bethesda, MD, USA), and the values were averaged (5–10 measurements per image, n = 7 mice, 200×).

### 4.5. Measurement of Fecal Parameters

Fecal pellets from each mouse were separately collected to calculate the fecal parameters. We collected the feces between 14:00 and 17:00. Each mouse was placed in a cage without food and water, and fresh fecal pellets were collected in 1.5 mL dry tubes immediately after excretion. After weighing, the fecal pellets were dried at 100 °C for 2 h. Fecal water content (%) = (wet weight − dry weight)/wet weight × 100%.

### 4.6. Measurement of Intestinal Transit

The mice were fasted for 12 h before performing the gastrointestinal motility test. The measurement of intestinal transit time was performed as previously described [47].

To measure the gastrointestinal transit time, 200 μL carbon solution (5% carbon in physiological saline) was provided to the mice via oral gavage. The mice were monitored for the excretion of the first black fecal pellet. The gastrointestinal transit time was the interval between gavaging and the first black feces excretion. 

To measure distal colon transit time, mice were anesthetized in a glass cylinder containing isoflurane (5%) and oxygen (100 mL/min) for 10–15 s. Then, a 3 mm diameter glass bead was placed into the mice colons (2 cm from the anal opening) using a glass rod. The time required to excrete the bead was considered to be the distal colon transit time.

To measure the small intestinal transit rate, carbon solution was provided to the mice via oral gavage, and their guts were removed 30 min after gavage. The small intestinal transit rate was the percentage of black carbon remaining in the small intestine (%).

### 4.7. Abdominal Withdrawal Reflex (AWR) Score

The visceral sensory responses to colorectal distention (CRD) were quantified via AWR scoring. A distension balloon catheter was placed in the descending colons of the mice as previously described [48]. CRD was controlled at 20, 40, 60, and 80 mmHg pressures. Variability of less than 20% response between 2 consecutive CRD tests at 60 mmHg was stable.

### 4.8. Colonic Smooth Muscle Contraction Test

The activity of colonic smooth muscle was recorded as previously described [49]. Colonic smooth muscle strips 1 cm in length and 0.3 cm in width were prepared and placed in individual 25 mL chambers on in organ bath system containing oxygenated (95% O_2_+ 5% CO_2_) Kreb’s solution (119 mmol/L NaCl, 4.7 mmol/L KCl, 25 mmol/L NaHCO_3_, 1.2 mmol/L NaH_2_PO_4_, 2.5 mmol/L CaCl_2_, 1.2 mmol/L MgSO_4_, and 11.1 mmol/L D-Glucose; pH 7.30–7.40) at 37 °C. The activity of each strip was recorded using a LabChartReader_8.1.13 multichannel physiological signal system. The strips were equilibrated for 30 min using a preload set at 1.0 g until they achieved a stable spontaneous contractile. Acetylcholine (Ach, Sigma, A6500) was added into the bath chamber to observe changes in muscle contraction.

The contractile amplitude, frequency, and motility index (MI) were recorded and calculated. The MI is the area under the contractile curve in unit time, which could be considered a comprehensive evaluation of muscle activity containing muscle amplitude and frequency.

### 4.9. Crypt Isolation and Organoid Culture

Crypt isolation and organoid culture were performed as previously described elsewhere with some modifications [50]. In brief, the distal colons were separated from the mice and cleaned of feces, fat, and blood vessels. The tissue was then washed 3 times with PBS containing Antibiotic-Antimycotic (AA, 15240-062, Gibco, Life Technologies, Carlsbad, CA, USA) and then 2 times with chelating buffer in 15 mL centrifuge tubes. Next, the tissue was transferred to a six-well plate, cut into 1–2 mm pieces, and digested in chelating buffer with 2.5 mM EDTA (E1170, Solarbio, Peking, China) using a shaker (150 rpm, 45 min). Then, the tissue was pipetted into a suspension (up and down and in an intense manner) with an FBS-coated 5 mL pipet 30–50 times. The crypts were obtained after filtering the solution through a 100 μm filter. Then, the crypts were centrifuged and washed 2 more times with chelating buffer. Then, 50 μL Matrigel (#356231, Corning, New York, NY, USA) was added to the crypt pellets (500 crypts per well) and suspended into a pre-warmed 24-well plate. The plate was incubated at 37 °C for 10 min before 400 μL IntestiCult Organoid Growth Medium (#06005, STEMCELL Technologies, Vancouver, BC, Canada) was added to it. The medium was also supplemented with 30 ng/mL Wnt3a. The plate was incubated at 37 °C and 5% CO_2_. The organoid medium was changed every three days. Images of the colonoids were captured and viewed using a microscope (Axio Observer 7, Carl Zeiss). The measurement of the colonoids was performed as previously described [51].

### 4.10. Immunofluorescence and EDU Staining in Colonoids

For immunofluorescence staining, the colonoids were collected from Matrigel with Cell Recovery Solution (Corning, #354253) and fixed with 4% PFA for 30 min. The next steps were the same as performed in the tissue sections (described above). The BeyoClick™ EDU Cell Proliferation Kit with Alexa Fluor 594 (C0078S, Beyotime Biotechnology, Shanghai, China) was used to evaluate cell proliferation in colonoids. Briefly, the colonoids were treated with EDU (10 μM) at 37 °C for 2 h and then fixed in 4% PFA at room temperature for 15 min. After permeabilization with 0.3% Triton for 15 min, the colonoids were reacted with reaction mixture fluid for 30 min. The nuclei were stained with DAPI for 10 min. The images of the colonoids were captured using a fluorescence microscope (Axio Observer 7, Carl Zeiss). The ratio of positive cells was calculated as previously described.

### 4.11. Cell Culture

The human colonic adenocarcinoma LS174T cell line (purchased from ScienCell, San Diego, Los Angeles, USA) was cultured in DMEM containing 10% FBS and 1% nonessential amino acids. The medium was replaced every other day. The LS174T cells were infected with lentivirus packaging *Piezo1*-siRNA (Genechem, Shanghai, China) to generate the Piezo1-knockdown LS174T cell line (Piezo1-KD); the protocol used was described in our previous study [27]. The LS174T cells infected with the lentivirus packaging control-siRNA were used as the control group in the RNA-seq analysis.

### 4.12. RT-PCR Analysis

RNA was extracted from the colon crypts and colonoids using Trizol reagent (R401-01, Vazyme, Nanjing, China) according to the manufacturer’s protocol. Complementary DNAs were obtained via reverse transcription using HiScript III RT SuperMix (Vazyme, R333-01). A real-time polymerase chain reaction (RT-PCR) was conducted using SYBR Green Transcription Master Mix (TaKaRa, Dalian, China) in a LightCycler 480 (Roche). The relative expression levels of the genes were normalized to *GAPDH*. The primer sequences used are shown in Table 1.

### 4.13. RNA-Seq and Bioinformatics Analysis

RNA from LS174T cells was extracted according to the manufacturer’s protocol. Reverse transcription was used to generate cDNA. After repair, the cDNA obtained from the previous step was amplified via PCR, and the products were purified using Ampure XP beads and subsequently dissolved into an EB solution. Then, the products were evaluated. The double-stranded PCR products from the previous step were heated, denatured, and circularized to obtain the final library. A DNA nanoball (DNB) was prepared to perform sequencing on the DNBSEQ-T7 platform (BGI Genomics). Differential gene analysis (DGE analysis) was performed using the DESeq2 (v1.4.5) [52]. Differentially expressed genes (DEGs) were selected by |Log2 (Fold Change)| > 1 and *p*-value < 0.05. To further analyze the results of the RNA-seq analysis, Kyoto Encyclopedia of Genes and Genomes (KEGG) analysis was performed to identify the significant pathways. The significance level for the terms and pathways was *p*-value < 0.05. The data provided in this study are available from the corresponding author upon request. The data are not publicly available because it contains elements of other studies that have not yet been completed.

### 4.14. Statistical Analysis

Statistical analysis graphs for each experiment were created using GraphPad Prism software (8.0). All data are shown as the mean  ±  SEM. A two-tailed unpaired *t*-test was used to analyze the statistically significant differences between the two groups. *p*-value < 0.05 was considered statistically significant.

## 5. Conclusions

In conclusion, our results demonstrate the importance of GC Piezo1 in intestinal transit, showing that it plays a role in regulating intestinal epithelial homeostasis. Our results provide a new perspective for explaining slowed intestinal transit, and we hope that GC Piezo1 can be a new therapeutic target in slow transit constipation. In the future, Piezo1^ΔGC^ mice may be used in animal models to study slow transit intestinal diseases.

## Figures and Tables

**Table 1:** Specific primers (mouse) used in RT-PCR analysis.

Gene	Forward (5′-3′)	Reverse (5′-3′)
*GAPDH*	AACAGCAACTCCCACTCTTC	CCTGTTGCTGTAGCCGTATT
*Piezo1*	TAACACCCTCTGTGTGTCATGGT	GGGAGGTCTACGAAGTTCTTGAG
*Mucin2*	ATGCCCACCTCCTCAAAGAC	GTAGTTTCCGTTGGAACAGTGAA
*Alpi*	GGCCATCTAGGACCGGAGA	TGTCCACGTTGTATGTCTTGG
*Chga*	CCAAGGTGATGAAGTGCGTC	GGTGTCGCAGGATAGAGAGGA
*cKit*	GACCCGACGCAACTTCCTTA	GAGCATCTTCACGGCAACTGT
*Ki67*	ATCATTGACCGCTCCTTTAGGT	GCTCGCCTTGATGGTTCCT
*Lgr5*	GACGCTGGGTTATTTCAAGTTCAA	CAGCCAGCTACCAAATAGGTGCTC
*Sox9*	GAGCCGGATCTGAAGAGGGA	GCTTGACGTGTGGCTTGTTC
*EphB2*	CCATTGAACAGGACTACAGACTACC	CACCGTGTTAAAGCTGGTGTAG
*β-catenin*	ATGGAGCCGGACAGAAAAGC	TGGGAGGTGTCAACATCTTCT
*Axin2*	GGACTGGGGAGCCTAAAGGT	AAGGAGGGACTCCATCTACGC
*Lrp5*	AAGGGTGCTGTGTACTGGAC	AGAAGAGAACCTTACGGGACG
*Hes1*	TCAACACGACACCGGACAAAC	ATGCCGGGAGCTATCTTTCTT
*Atoh1*	CAGGGTGAGCTGGTAAGGAG	GCCAAGCTCGTCCACTACA
*Dll1*	GCGACTGAGGTGTAAGATGGAA	TCTCAGCAGCATTCATCGGG
*Dll4*	TTCCAGGCAACCTTCTCCGA	ACTGCCGCTATTCTTGTCCC
*Yap1*	CGCTCTTCAATGCCGTCATG	AGTCATGGCTTGCTCCCATC
*Foxo1*	AGGATAAGGGCGACAGCAAC	CTTGCCTCCCTCTGGATTGA
*Foxo3*	TTGGTCAATCAGAACTTGCT	CCCATGTTGCTGACAGAAT
*Foxo4*	GGTGCCCTACTTCAAGGACA	AGCTTGCTGCTGCTATCCAT

## Data Availability

All data associated with this study are present in the text or the Supplementary Materials.

## Supplementary Materials

The following supporting information can be downloaded at: https://www.mdpi.com/article/10.3390/ijms241814377/s1.

## References

[1] Camilleri M., Ford A.C., Mawe G.M., Dinning P.G., Rao S.S., Chey W.D., Simren M., Lembo A., Young-Fadok T.M., Chang L. (2017). Chronic constipation. Nat. Rev. Dis. Primers.

[2] Xu Y., Xiong Y., Liu Y., Li G., Bai T., Zheng G., Hou X., Song J. (2023). Activation of goblet cell Piezo1 alleviates mucus barrier damage in mice exposed to WAS by inhibiting H3K9me3 modification. Cell Biosci..

[3] Parthasarathy G., Chen J., Chen X., Chia N., O’Connor H.M., Wolf P.G., Gaskins H.R., Bharucha A.E. (2016). Relationship Between Microbiota of the Colonic Mucosa vs Feces and Symptoms, Colonic Transit, and Methane Production in Female Patients With Chronic Constipation. Gastroenterology.

[4] Heitmann P.T., Vollebregt P.F., Knowles C.H., Lunniss P.J., Dinning P.G., Scott S.M. (2021). Understanding the physiology of human defaecation and disorders of continence and evacuation. Nat. Rev. Gastroenterol. Hepatol..

[5] Paone P., Cani P.D. (2020). Mucus barrier, mucins and gut microbiota: The expected slimy partners?. Gut.

[6] Bergstrom K., Shan X., Casero D., Batushansky A., Lagishetty V., Jacobs J.P., Hoover C., Kondo Y., Shao B., Gao L. (2020). Proximal colon-derived O-glycosylated mucus encapsulates and modulates the microbiota. Science.

[7] Yang P.J., LaMarca M., Kaminski C., Chu D.I., Hu D.L. (2017). Hydrodynamics of defecation. Soft Matter.

[8] Gao C.C., Li G.W., Wang T.T., Gao L., Wang F.F., Shang H.W., Yang Z.J., Guo Y.X., Wang B.Y., Xu J.D. (2021). Rhubarb extract relieves constipation by stimulating mucus production in the colon and altering the intestinal flora. Biomed. Pharm..

[9] Jiang Y., Yang X., Jiang J., Xiao B. (2021). Structural Designs and Mechanogating Mechanisms of the Mechanosensitive Piezo Channels. Trends Biochem. Sci..

[10] Mercado-Perez A., Beyder A. (2022). Gut feelings: Mechanosensing in the gastrointestinal tract. Nat. Rev. Gastroenterol. Hepatol..

[11] Retailleau K., Duprat F., Arhatte M., Ranade S.S., Peyronnet R., Martins J.R., Jodar M., Moro C., Offermanns S., Feng Y. (2015). Piezo1 in Smooth Muscle Cells Is Involved in Hypertension-Dependent Arterial Remodeling. Cell Rep..

[12] Wang L., You X., Lotinun S., Zhang L., Wu N., Zou W. (2020). Mechanical sensing protein PIEZO1 regulates bone homeostasis via osteoblast-osteoclast crosstalk. Nat. Commun..

[13] Solis A.G., Bielecki P., Steach H.R., Sharma L., Harman C.C.D., Yun S., de Zoete M.R., Warnock J.N., To S.D.F., York A.G. (2019). Author Correction: Mechanosensation of cyclical force by PIEZO1 is essential for innate immunity. Nature.

[14] Geng J., Shi Y., Zhang J., Yang B., Wang P., Yuan W., Zhao H., Li J., Qin F., Hong L. (2021). TLR4 signalling via Piezo1 engages and enhances the macrophage mediated host response during bacterial infection. Nat. Commun..

[15] Koser D.E., Thompson A.J., Foster S.K., Dwivedy A., Pillai E.K., Sheridan G.K., Svoboda H., Viana M., Costa L.D., Guck J. (2016). Mechanosensing is critical for axon growth in the developing brain. Nat. Neurosci..

[16] Zhu W., Hou F., Fang J., Bahrani Fard M.R., Liu Y., Ren S., Wu S., Qi Y., Sui S., Read A.T. (2021). The role of Piezo1 in conventional aqueous humor outflow dynamics. iScience.

[17] Sun W., Chi S., Li Y., Ling S., Tan Y., Xu Y., Jiang F., Li J., Liu C., Zhong G. (2019). The mechanosensitive Piezo1 channel is required for bone formation. eLife.

[18] Romac J.M., Shahid R.A., Swain S.M., Vigna S.R., Liddle R.A. (2018). Piezo1 is a mechanically activated ion channel and mediates pressure induced pancreatitis. Nat. Commun..

[19] Yu J.L., Liao H.Y. (2021). Piezo-type mechanosensitive ion channel component 1 (Piezo1) in human cancer. Biomed. Pharm..

[20] Gustafsson J.K., Johansson M.E.V. (2022). The role of goblet cells and mucus in intestinal homeostasis. Nat. Rev. Gastroenterol. Hepatol..

[21] Liu Y., Fang F., Xiong Y., Wu J., Li X., Li G., Bai T., Hou X., Song J. (2022). Reprogrammed fecal and mucosa-associated intestinal microbiota and weakened mucus layer in intestinal goblet cell—Specific Piezo1-deficient mice. Front. Cell Infect. Microbiol..

[22] Rothenberg M.E., Nusse Y., Kalisky T., Lee J.J., Dalerba P., Scheeren F., Lobo N., Kulkarni S., Sim S., Qian D. (2012). Identification of a cKit(+) colonic crypt base secretory cell that supports Lgr5(+) stem cells in mice. Gastroenterology.

[23] Houtekamer R.M., van der Net M.C., Maurice M.M., Gloerich M. (2022). Mechanical forces directing intestinal form and function. Curr. Biol..

[24] Gudipaty S.A., Lindblom J., Loftus P.D., Redd M.J., Edes K., Davey C.F., Krishnegowda V., Rosenblatt J. (2017). Mechanical stretch triggers rapid epithelial cell division through Piezo1. Nature.

[25] Ludikhuize M.C., Meerlo M., Gallego M.P., Xanthakis D., Burgaya Julia M., Nguyen N.T.B., Brombacher E.C., Liv N., Maurice M.M., Paik J.H. (2020). Mitochondria Define Intestinal Stem Cell Differentiation Downstream of a FOXO/Notch Axis. Cell Metab..

[26] Beumer J., Clevers H. (2021). Cell fate specification and differentiation in the adult mammalian intestine. Nat. Rev. Mol. Cell Biol..

[27] Xu Y., Bai T., Xiong Y., Liu C., Liu Y., Hou X., Song J. (2021). Mechanical stimulation activates Piezo1 to promote mucin2 expression in goblet cells. J. Gastroenterol. Hepatol..

[28] Tao Y., Qiao S.M., Lv C.J., Yun X.M., Yue M.F., Fang Y.L., Wei Z.F., Dai Y., Xia Y.F. (2022). Phytoestrogen arctigenin preserves the mucus barrier in inflammatory bowel diseases by inhibiting goblet cell apoptosis via the ERbeta/TRIM21/PHB1 pathway. Phytother Res..

[29] Chen L., Jiao T., Liu W., Luo Y., Wang J., Guo X., Tong X., Lin Z., Sun C., Wang K. (2022). Hepatic cytochrome P450 8B1 and cholic acid potentiate intestinal epithelial injury in colitis by suppressing intestinal stem cell renewal. Cell Stem. Cell.

[30] Eisenhoffer G.T., Loftus P.D., Yoshigi M., Otsuna H., Chien C.B., Morcos P.A., Rosenblatt J. (2012). Crowding induces live cell extrusion to maintain homeostatic cell numbers in epithelia. Nature.

[31] Duckworth C.A. (2021). Identifying key regulators of the intestinal stem cell niche. Biochem. Soc. Trans..

[32] Huelsz-Prince G., Kok R.N.U., Goos Y., Bruens L., Zheng X., Ellenbroek S., Van Rheenen J., Tans S., van Zon J.S. (2022). Mother cells control daughter cell proliferation in intestinal organoids to minimize proliferation fluctuations. eLife.

[33] Zhang X., Yang H., Zheng J., Jiang N., Sun G., Bao X., Lin A., Liu H. (2021). Chitosan oligosaccharides attenuate loperamide-induced constipation through regulation of gut microbiota in mice. Carbohydr. Polym..

[34] Ermund A., Schutte A., Johansson M.E., Gustafsson J.K., Hansson G.C. (2013). Studies of mucus in mouse stomach, small intestine, and colon. I. Gastrointestinal mucus layers have different properties depending on location as well as over the Peyer’s patches. Am. J. Physiol. Gastrointest. Liver Physiol..

[35] Wang J.K., Wei W., Zhao D.Y., Wang H.F., Zhang Y.L., Lei J.P., Yao S.K. (2022). Intestinal mucosal barrier in functional constipation: Dose it change?. World J. Clin. Cases.

[36] Pellegrinet L., Rodilla V., Liu Z., Chen S., Koch U., Espinosa L., Kaestner K.H., Kopan R., Lewis J., Radtke F. (2011). Dll1- and dll4-mediated notch signaling are required for homeostasis of intestinal stem cells. Gastroenterology.

[37] Hong A.W., Meng Z., Guan K.L. (2016). The Hippo pathway in intestinal regeneration and disease. Nat. Rev. Gastroenterol. Hepatol..

[38] Ward D., Montes Olivas S., Fletcher A., Homer M., Marucci L. (2020). Cross-talk between Hippo and Wnt signalling pathways in intestinal crypts: Insights from an agent-based model. Comput. Struct. Biotechnol. J..

[39] Zhou D., Zhang Y., Wu H., Barry E., Yin Y., Lawrence E., Dawson D., Willis J.E., Markowitz S.D., Camargo F.D. (2011). Mst1 and Mst2 protein kinases restrain intestinal stem cell proliferation and colonic tumorigenesis by inhibition of Yes-associated protein (Yap) overabundance. Proc. Natl. Acad. Sci. USA.

[40] Gopinath S.D., Webb A.E., Brunet A., Rando T.A. (2014). FOXO3 promotes quiescence in adult muscle stem cells during the process of self-renewal. Stem. Cell Rep..

[41] Kim D.Y., Hwang I., Muller F.L., Paik J.H. (2015). Functional regulation of FoxO1 in neural stem cell differentiation. Cell Death Differ..

[42] Ludikhuize M.C., Rodriguez Colman M.J. (2021). Metabolic Regulation of Stem Cells and Differentiation: A Forkhead Box O Transcription Factor Perspective. Antioxid Redox Signal.

[43] Zhong G., Su S., Li J., Zhao H., Hu D., Chen J., Li S., Lin Y., Wen L., Lin X. (2023). Activation of Piezo1 promotes osteogenic differentiation of aortic valve interstitial cell through YAP-dependent glutaminolysis. Sci. Adv..

[44] Daoud F., Holmberg J., Alajbegovic A., Grossi M., Rippe C., Sward K., Albinsson S. (2021). Inducible Deletion of YAP and TAZ in Adult Mouse Smooth Muscle Causes Rapid and Lethal Colonic Pseudo-Obstruction. Cell. Mol. Gastroenterol. Hepatol..

[45] Sugisawa E., Takayama Y., Takemura N., Kondo T., Hatakeyama S., Kumagai Y., Sunagawa M., Tominaga M., Maruyama K. (2020). RNA Sensing by Gut Piezo1 Is Essential for Systemic Serotonin Synthesis. Cell.

[46] Yoshimoto S., Taguchi M., Sumi S., Oka K., Okamura K. (2023). Establishment of a novel protocol for formalin-fixed paraffin-embedded organoids and spheroids. Biol. Open.

[47] Israelyan N., Del Colle A., Li Z., Park Y., Xing A., Jacobsen J.P.R., Luna R.A., Jensen D.D., Madra M., Saurman V. (2019). Effects of Serotonin and Slow-Release 5-Hydroxytryptophan on Gastrointestinal Motility in a Mouse Model of Depression. Gastroenterology.

[48] Wang J., Zhao D., Lei Z., Ge P., Lu Z., Chai Q., Zhang Y., Qiang L., Yu Y., Zhang X. (2023). TRIM27 maintains gut homeostasis by promoting intestinal stem cell self-renewal. Cell. Mol. Immunol..

[49] Zhang L., Wang R., Bai T., Xiang X., Qian W., Song J., Hou X. (2019). EphrinB2/ephB2-mediated myenteric synaptic plasticity: Mechanisms underlying the persistent muscle hypercontractility and pain in postinfectious IBS. FASEB J..

[50] Mahe M.M., Sundaram N., Watson C.L., Shroyer N.F., Helmrath M.A. (2015). Establishment of human epithelial enteroids and colonoids from whole tissue and biopsy. J. Vis. Exp. (JoVE).

[51] Lindemans C.A., Calafiore M., Mertelsmann A.M., O’Connor M.H., Dudakov J.A., Jenq R.R., Velardi E., Young L.F., Smith O.M., Lawrence G. (2015). Interleukin-22 promotes intestinal-stem-cell-mediated epithelial regeneration. Nature.

[52] Love M.I., Huber W., Anders S. (2014). Moderated estimation of fold change and dispersion for RNA-seq data with DESeq2. Genome Biol..

