# Supplementary material for: Slowed Intestinal Transit Induced by Less Mucus in Intestinal Goblet Cell Piezo1-Deficient Mice through Impaired Epithelial Homeostasis
Source: Int J Mol Sci. 2023 Sep 21;24(18):14377. doi: 10.3390/ijms241814377 (PMC10531822; doi:10.3390/ijms241814377)
Supplement: Supplementary file 1 [file ijms-24-14377-s001.zip › ijms-2619413-supplementary.pdf]

Supplementary data

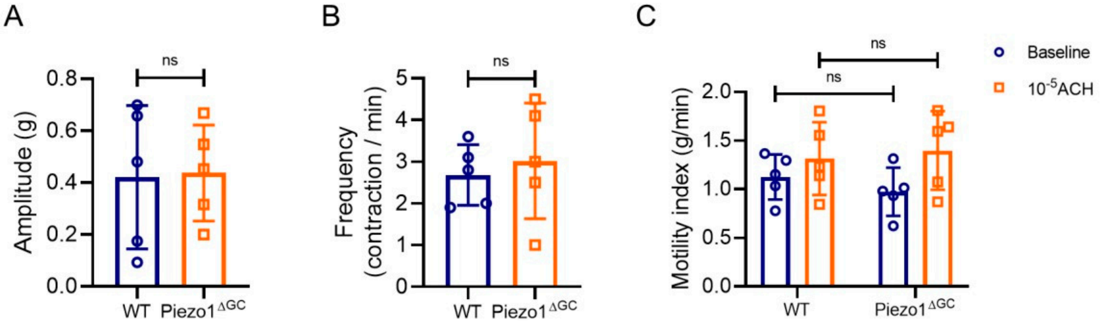

Figure S1. Spontaneous activities and acetylcholine-induced responses in colonic smooth muscles from WT and Piezo1$^{\Delta GC}$ mice. (**A**) Contractile amplitude. (**B**) Contractile frequency. (**C**) Motility index (MI) at baseline and after acetylcholine activation. The MI is the area under the contractile curve in unit time. At least three independent experiments were conducted. Data are presented as the mean ± SEM. (n = 5 mice). ns, not significant.
